# Risk factors, prognostic factors, and nomograms for bone metastasis in patients with newly diagnosed infiltrating duct carcinoma of the breast: a population-based study

**DOI:** 10.1186/s12885-020-07635-1

**Published:** 2020-11-25

**Authors:** Zhangheng Huang, Chuan Hu, Kewen Liu, Luolin Yuan, Yinglun Li, Chengliang Zhao, Chanchan Hu

**Affiliations:** 1 Department of Spine Surgery, Affiliated Hospital of Chengde Medical University, No.36 Nanyingzi St, Shuangqiao District, Chengde, Hebei Province China; 2 Department of Orthopedic, The Affiliated Hospital of Qingdao University, Shinan District, Qingdao, Shandong Province China; 3 Department of Oncology, Affiliated Hospital of Chengde Medical University, No.36 Nanyingzi St, Shuangqiao District, Chengde, Hebei Province China

**Keywords:** Breast cancer, Infiltrating duct carcinoma, Bone metastasis, Predictor, Prognosis, Nomogram

## Abstract

**Background:**

Breast cancer is the most common malignancy in women, and it is also the leading cause of death in female patients; the most common pathological type of BC is infiltrating duct carcinoma (IDC). Some nomograms have been developed to predict bone metastasis (BM) in patients with breast cancer. However, there are no studies on diagnostic and prognostic nomograms for BM in newly diagnosed IDC patients.

**Methods:**

IDC patients with newly diagnosed BM from 2010 to 2016 in the Surveillance, Epidemiology and End Results (SEER) database were reviewed. Multivariate logistic regression analysis was used to identify risk factors for BM in patients with IDC. Univariate and multivariate Cox proportional hazards regression analysis were used to explore the prognostic factors of BM in patients with IDC. We then constructed nomograms to predict the risk and prognosis of BM for patients with IDC. The results were validated using bootstrap resampling and retrospective research on 113 IDC patients with BM from 2015 to 2018 at the Affiliated Hospital of Chengde Medical University.

**Results:**

This study included 141,959 patients diagnosed with IDC in the SEER database, of whom 2383 cases were IDC patients with BM. The risk factors for BM in patients with IDC included sex, primary site, grade, T stage, N stage, liver metastasis, race, brain metastasis, breast cancer subtype, lung metastasis, insurance status, and marital status. The independent prognostic factors were brain metastases, race, grade, surgery, chemotherapy, age, liver metastases, breast cancer subtype, insurance status, and marital status. Through calibration, receiver operating characteristic curve and decision curve analyses, we found that the nomogram for predicting the prognosis of IDC patients with BM displayed great performance both internally and externally.

**Conclusion:**

These nomograms are expected to be a precise and personalized tool for predicting the risk and prognosis for BM in patients with IDC. This will help clinicians develop more rational and effective treatment strategies.

## Background

Breast cancer (BC) is the most common malignancy and the leading cause of death among all female cancer patients [1, 2]. Globally, there were approximately 2.1 million newly diagnosed female BC cases in 2018 [3]. Recently, with the advancement of early diagnosis and comprehensive treatment, the mortality rate of BC has gradually decreased, and distant metastasis has become the main cause of death for these patients [4, 5]. It has been reported that the incidence of metastases in BC patients ranges from 20 to 30% [6]. More importantly, bone metastasis (BM) accounts for 50% of all distant metastases in these patient [7]. At present, most BC patients with BM receive palliative treatment [8]. Although some patients choose surgery, it is not suitable for patients with multiple metastases or a poor overall health [9]. Some studies have shown that the median survival for patients with breast cancer and BM is only 24–36 months [10].

The TNM staging system is the most common tool used to predict the prognosis of cancer patients by assessing tumor size and location (T), distant metastasis (M), and regional lymph node metastasis (N) [11]. However, the TNM staging system does not sufficiently cover cancer biology or predict the outcome for all subtypes of BC [12]. In particular, the TNM staging system fails to quantify the risk for patients with distant metastatic malignancies. Therefore, an increasing number of cancer-related nomograms (statistical tools to estimate the probability of survival or a specific result through a simple graphical representation) have been developed for predicting the prognosis of cancer patients [13]. Nomograms have a number of advantages in predicting the prognosis of some malignant tumors compared to the traditional American Joint Committee for Cancer (AJCC) TNM staging system, making them a good alternative.

It is well established that histological subtypes of breast cancer affect prognosis, and the most common pathological type of BC is infiltrating duct carcinoma (IDC) [14]. At present, there are no studies that have focused on diagnostic and prognostic nomograms for BM in newly diagnosed IDC patients. Therefore, it is necessary to fully understand the epidemiological characteristics of IDC patients with BM to identify the risk and prognostic factors for BM. Well-developed clinical nomograms can be used to predict individual outcomes, which is beneficial to both patients and clinicians [15].

Thus, the aim of this study was to develop a predictive model by analyzing the data of the Surveillance, Epidemiology and End Results (SEER) database to determine the risk and prognosis for BM in patients with IDC.

## Methods

### Patients

We included patients with newly diagnosed IDC in the SEER database from 2010 to 2016 in our study. Exclusion criteria were as follows: (1) patients with two or more primary malignancies; (2) patients whose pathological type was not IDC; (3) patients missing important clinical pathological information, including laterality, primary tumor site, grade, TNM stage, or estrogen receptor (ER) or progesterone receptor (PR) status, or HER2 status. Finally, 141,959 patients diagnosed with IDC were included in the present study, of whom 2383 patients (1.68%) had BM, while 139,576 patients (98.32%) did not. In addition, we retrospectively collected data for IDC patients with BM from the Affiliated Hospital of Chengde Medical University (AHOCMU) between 2015 and 2018 as an external validation cohort for our research.

### Data collection

The variables were selected to identify the risk factors of BM in IDC patients are as follows: age at diagnosis, sex, race, tumor site, laterality, grade, T stage, N stage, liver metastasis, brain metastasis, lung metastasis, breast cancer subtype, ER status, PR status, HER2 status, insurance, and marital status. In our research, we also performed the survival analyses to study the prognostic factors of IDC patients with BM. In addition to the above variables, the treatment information, including surgery, radiotherapy, and chemotherapy, were also included to study the prognostic factors. Moreover, patients with overall survival (OS) less than 1 month were also excluded from the survival analyses. In the survival analysis, the main endpoint of our study was OS, which was defined as the date from diagnosis to death (due to any cause) or the date of the last follow-up. Risk of developing metastasis was defined as the risk of bone metastasis when the patient was first diagnosed with IDC of the breast. Survival prognosis was defined as the OS of the patient who was first diagnosed with IDC of the breast. Our study was approved by the Institutional Research Committee from AHOCMU.

### Development of a diagnostic nomogram

All statistical analyses in our research were performed in R software (version 3.6.1). To identify the risk factors of BM in IDC patients, univariate analysis was performed. Comparisons of continuous data were performed by independent t-tests, while the chi-square test or the Fisher exact probability method were used for categorical data. Variables with a *P* value < 0.05 in the univariate analysis were included in the multivariate logistic analysis to identify the risk factors for BM in IDC patients. Based on independent risk factors, the rms package was used to build a nomogram and calculate the individual risk score. Meanwhile, the receiver operating characteristic (ROC) curve was plotted, and the area under the curve (AUC) was used to show the discrimination of the nomogram. Moreover, a calibration curve and decision curve analyses (DCA) were performed to evaluate the nomogram [16].

### Development of a prognostic nomogram

To identify the prognostic factors of IDC patients with BM, 2383 patients were included to perform survival analyses. All BM patients were randomly divided into training (*n* = 1671) and validation (*n* = 712) cohorts with a ratio of 7:3. The classification process was completely randomized and it was performed in R software. The best age cutoff values for OS were determined by X-tile software; patients were divided into high, middle, and low groups. We then performed univariate Cox proportional hazards regression analysis to determine the OS-related variables. Afterward, significant variables in the univariate Cox proportional hazards regression analyses were incorporated into the multivariate Cox proportional hazards regression analyses to determine the independent prognostic factors for IDC patients with BM. Then, a nomogram based on the independent prognostic factors was established to predict the OS for IDC patients with BM. Additionally, time-dependent ROC curves of 1, 3, and 5 years were generated, and the corresponding time-dependent AUCs were used to show the discrimination of the nomogram. Calibration curves and DCA of 1, 3, and 5 years were established. To further validate that the nomogram could perform well in an independent cohort, we validated the nomogram with data from the SEER validation cohort and the AHOCMU cohort. Time-dependent ROC curve, calibration curve, and DCA were also performed in the validation cohort. In the present study, a *P* value< 0.05(two side) was identified as statistical significance.

## Results

### Baseline characteristics of the study population

Based on our criteria, a total of 141,959 IDC patients from the SEER database were included, and an additional 113 IDC patients with BM were identified from the AHOCMU for this study. Additionally, 1671 patients were included in the training cohort and 712 patients were included in the validation cohort. As shown in Table 1, 99.24% of the patients were female and 80.23% were white. The most common tumor grade of differentiation was grade II (42.01%). The most common primary site location was in the upper-outer quadrant of the breast (39.04%). There was minimal laterality, with left primary site origins accounting for 50.54% of the study group and right primary site origins accounting for 49.46%. The most common T and N stages were T1 (63.60%) and N0 (69.79%). Regarding the classifications of breast cancer subtypes, luminal A (HR+/HER2-) accounted for 71.09%. A total of 1133 (0.80%) patients had lung metastases, 202 (0.14%) patients had brain metastasis and 979 (0.69%) patients had liver metastases. Most patients were insured (98.10%) and married (62.57%). In our study, most patients were positive for PR (71.76%) and ER (81.51%). Regarding therapy, 136,494 (96.15%) of the patients underwent surgery, 61,831 (43.56%) underwent chemotherapy, and 80,424 (56.65%) underwent radiotherapy. Table 2 displays information on the clinical and pathological features for the IDC patients with BM.

**Table 1:** Clinical and pathological features of patients newly diagnosed as infiltrating duct carcinoma of breast

Variables	SEER (*N* = 141,959)	Percent
Ag		
22–54	49,438	34.83
55–79	81,114	57.14
≥ 80	11,407	8.03
Race		
White	113,888	80.23
Black	14,466	10.19
Other	13,605	9.58
Sex		
Female	140,883	99.24
Male	1076	0.76
Primary Site		
Nipple	479	0.34
Central portion of breast	7319	5.16
Upper-inner quadrant of breast	20,520	14.45
Lower-inner quadrant of breast	9267	6.53
Upper-outer quadrant of breast	55,426	39.04
Lower-outer quadrant of breast	12,097	8.52
Axillary tail of breast	736	0.52
Overlapping lesion of breast	36,115	25.44
Grade		
I	31,092	21.90
II	59,639	42.01
III	50,920	35.87
IV	308	0.22
Laterality		
Left - origin of primary	71,742	50.54
Right - origin of primary	70,217	49.46
T stage		
T1	90,286	63.60
T2	42,097	29.65
T3	6131	4.32
T4	3445	2.43
N stage		
N0	99,074	69.79
N1	32,876	23.16
N2	6570	4.63
N3	3439	2.42
Radiotherapy		
No	61,535	43.35
Yes	80,424	56.65
Chemotherapy		
No	80,128	56.44
Yes	61,831	43.56
Surgery		
No	5465	3.85
Yes	136,494	96.15
Brain metastasis		
No	141,757	99.86
Yes	202	0.14
Liver metastasis		
No	140,980	99.31
Yes	979	0.69
Lung metastasis		
No	140,826	99.20
Yes	1133	0.80
Breast subtype		
HR−/HER2-	17,731	12.49
VHR−/HER2+	6900	4.86
HR+/HER2-	100,919	71.09
HR+/HER2+	16,409	11.56
ER status		
Negative	26,250	18.49
Positive	115,709	81.51
PR status		
Negative	40,088	28.24
Positive	101,871	71.76
HER2 status		
Negative	118,650	83.58
Positive	23,309	16.42
Insurance		
Uninsured	2701	1.90
Insured	139,258	98.10
Marital status		
Unmarried	53,130	37.43
Married	88,829	62.57

**Table 2:** Clinical and pathological features of patients newly diagnosed as infiltrating duct carcinoma with bone metastasis

Variables	Total cohort		Training cohort		Validation cohort	
	*N* = 2383		*N* = 1671		*N* = 712	
	n	%	n	%	n	%
Age						
22–54	851	35.71	596	35.67	255	35.81
55–79	1294	54.30	901	53.92	393	55.20
≥ 80	238	9.99	174	10.41	64	8.99
Race						
Black	317	13.30	228	13.65	89	12.50
Other	187	7.85	123	7.36	64	8.99
White	1879	78.85	1320	78.99	559	78.51
Sex						
Female	2341	98.24	1638	98.03	703	98.74
Male	42	1.76	33	1.97	9	1.26
Primary Site						
Nipple	13	0.55	12	0.72	1	0.14
Central portion of breast	223	9.36	163	9.75	60	8.43
Upper-inner quadrant of breast	229	9.61	170	10.17	59	8.29
Lower-inner quadrant of breast	156	6.55	112	6.70	44	6.18
Upper-outer quadrant of breast	865	36.30	603	36.09	262	36.80
Lower-outer quadrant of breast	184	7.72	133	7.96	51	7.16
Axillary tail of breast	19	0.80	10	0.60	9	1.26
Overlapping lesion of breast	694	29.12	468	28.01	226	31.74
Grade						
I	156	6.55	106	6.34	50	7.02
II	1104	46.33	785	46.98	319	44.80
III	1118	46.91	777	46.50	341	47.90
IV	5	0.21	3	0.18	2	0.28
Laterality						
Left - origin of primary	1247	52.33	869	52.00	378	53.09
Right - origin of primary	1136	47.67	802	48.00	334	46.91
T stage						
T1	336	14.10	253	15.14	83	11.66
T2	987	41.42	670	40.10	317	44.52
T3	405	16.99	291	17.41	114	16.01
T4	655	27.49	457	27.35	198	27.81
N stage						
N0	557	23.37	380	22.74	177	24.86
N1	1160	48.68	820	49.07	340	47.75
N2	321	13.47	225	13.47	96	13.48
N3	345	14.48	246	14.72	99	13.91
Radiotherapy						
No	1782	74.78	1239	74.15	543	76.26
Yes	601	25.22	432	25.85	169	23.74
Chemotherapy						
No	976	40.96	688	41.17	288	40.45
Yes	1407	59.04	983	58.83	424	59.55
Surgery						
No	1465	61.48	1018	60.92	447	62.78
Yes	918	38.52	653	39.08	265	37.22
Brain metastasis						
No	2260	94.84	1582	94.67	678	95.22
Yes	123	5.16	89	5.33	34	4.78
Liver metastasis						
No	1881	78.93	1338	80.07	543	76.26
Yes	502	21.07	333	19.93	169	23.74
Lung metastasis						
No	1811	76.00	1258	75.28	553	77.67
Yes	572	24.00	413	24.72	159	22.33
Breast subtype						
HR−/HER2-	238	9.99	168	10.05	70	9.83
HR−/HER2+	167	7.01	116	6.94	51	7.16
HR+/HER2-	1525	63.99	1091	65.29	434	60.96
HR+/HER2+	453	19.01	296	17.72	157	22.05
HER2 status						
Negative	1763	73.98	1259	75.34	504	70.79
Positive	620	26.02	412	24.66	208	29.21
Insurance						
Uninsured	126	5.29	90	5.39	36	5.06
Insured	2257	94.71	1581	94.61	676	94.94
Marital status						
Unmarried	1101	46.20	760	45.48	341	47.89
Married	1282	53.80	911	54.52	371	52.11

### Risk factors for IDC patients with BM

As shown in Table 3, variables with a *P* value < 0.05 in the univariate analysis were included in the multivariate logistic regression analysis to determine the risk factors for BM in IDC patients. The results revealed that sex, primary site, grade, T stage, N stage, brain metastasis, lung metastasis, liver metastasis, breast cancer subtype, race, insurance status, and marital status were independent predictors for BM in IDC patients (Table 4).

**Table 3:** Univariate analysis of risk factor of bone metastasis in infiltrating duct carcinoma patients

Variable	Without bone metastasis number (n)	With bone metastasis number (n)	Chi-square	*P*-value
Age				
22–54	48,587	851	1.447	0.148
55–79	79,820	1294		
≥ 80	11,169	238		
Race				
Black	14,149	317	31.319	< 0.001
Other	13,418	187		
White	112,009	1879		
Sex				
Female	138,542	2341	32.512	< 0.001
Male	1034	42		
Primary Site				
Nipple	466	13	148.540	< 0.001
Central portion of breast	7096	223		
Upper-inner quadrant of breast	20,291	229		
Lower-inner quadrant of breast	9111	156		
Upper-outer quadrant of breast	54,561	865		
Lower-outer quadrant of breast	11,913	184		
Axillary tail of breast	717	19		
Overlapping lesion of breast	35,421	694		
Grade				
I	30,963	156	354.137	< 0.001
II	58,535	1104		
III	49,802	1118		
IV	303	5		
Laterality				
Left - origin of primary	70,495	1247	3.113	0.078
Right - origin of primary	69,081	1136		
T stage				
T1	89,950	336	8220.550	< 0.001
T2	41,110	987		
T3	5726	405		
T4	2790	655		
N stage				
N0	98,517	557	3293.151	< 0.001
N1	31,716	1160		
N2	6249	321		
N3	3094	345		
Brain metastasis				
No	139,497	2260		< 0.001
Yes	79	123		
Liver metastasis				
No	139,099	1881	14,692.994	< 0.001
Yes	477	502		
Lung metastasis				
No	139,015	1811	16,483.956	< 0.001
Yes	561	572		
Breast subtype				
HR−/HER2-	17,493	238	168.712	< 0.001
HR−/HER2+	6733	167		
HR+/HER2-	99,394	1525		
HR+/HER2+	15,956	453		
ER status				
Negative	25,818	432	0.212	0.645
Positive	113,758	1951		
PR status				
Negative	39,323	765	17.850	< 0.001
Positive	100,253	1618		
HER2 status				
Negative	116,887	1763	162.697	< 0.001
Positive	22,689	620		
Insurance				
Uninsured	2575	126	148.772	< 0.001
Insured	137,001	2257		
Marital status				
Unmarried	52,029	1101	79.707	< 0.001
Married	87,547	1282

**Table 4:** Multivariate logistic regression analysis of risk factor of bone metastasis in infiltrating duct carcinoma patients

Variables	Multivariate logistic regression analysis	
	HR (95% CI)	*P* value
Sex		
Female	Reference	
Male	1.507 (1.052–2.159)	0.025
Primary Site		
Nipple	Reference	
Central portion of breast	1.169 (0.612–2.231)	0.636
Upper-inner quadrant of breast	1.062 (0.555–2.031)	0.856
Lower-inner quadrant of breast	1.442 (0.748–2.783)	0.275
Upper-outer quadrant of breast	1.084 (0.573–2.047)	0.805
Lower-outer quadrant of breast	1.033 (0.538–1.985)	0.922
Axillary tail of breast	2.024 (0.900–4.552)	0.088
Overlapping lesion of breast	1.178 (0.623–2.228)	0.614
Grade		
I	Reference	
II	1.801 (1.498–2.165)	< 0.001
III	1.266 (1.043–1.537)	0.017
IV	0.422 (0.127–1.402)	0.159
T stage		
T1	Reference	
T2	4.015 (3.499–4.607)	< 0.001
T3	7.638 (6.417–9.091)	< 0.001
T4	17.022 (14.330–20.218)	< 0.001
N stage		
N0	Reference	
N1	2.709 (2.408–3.047)	< 0.001
N2	2.570 (2.174–3.038)	< 0.001
N3	4.651 (3.912–5.529)	< 0.001
Brain metastasis		
No	Reference	
Yes	14.890 (10.102–21.947)	< 0.001
Liver metastasis		
No	Reference	
Yes	19.038 (16.042–22.593)	< 0.001
Lung metastasis		
No	Reference	
Yes	13.368 (11.400–15.675)	< 0.001
Breast Subtype		
HR−/HER2-	Reference	
HR−/HER2+	1.201 (0.938–1.539)	0.146
HR+/HER2-	2.496 (2.096–2.972)	< 0.001
HR+/HER2+	2.289 (1.886–2.778)	< 0.001
Race		
Black	Reference	0.005
Other	0.811 (0.655–1.005)	0.055
White	1.073 (0.928–1.240)	0.341
Insurance		
Uninsured	Reference	
Insured	0.686 (0.548–0.859)	0.001
Marital status		
Unmarried	Reference	
Married	0.861 (0.782–0.947)	0.002

### Diagnostic nomogram development and validation

**Fig. 1:**
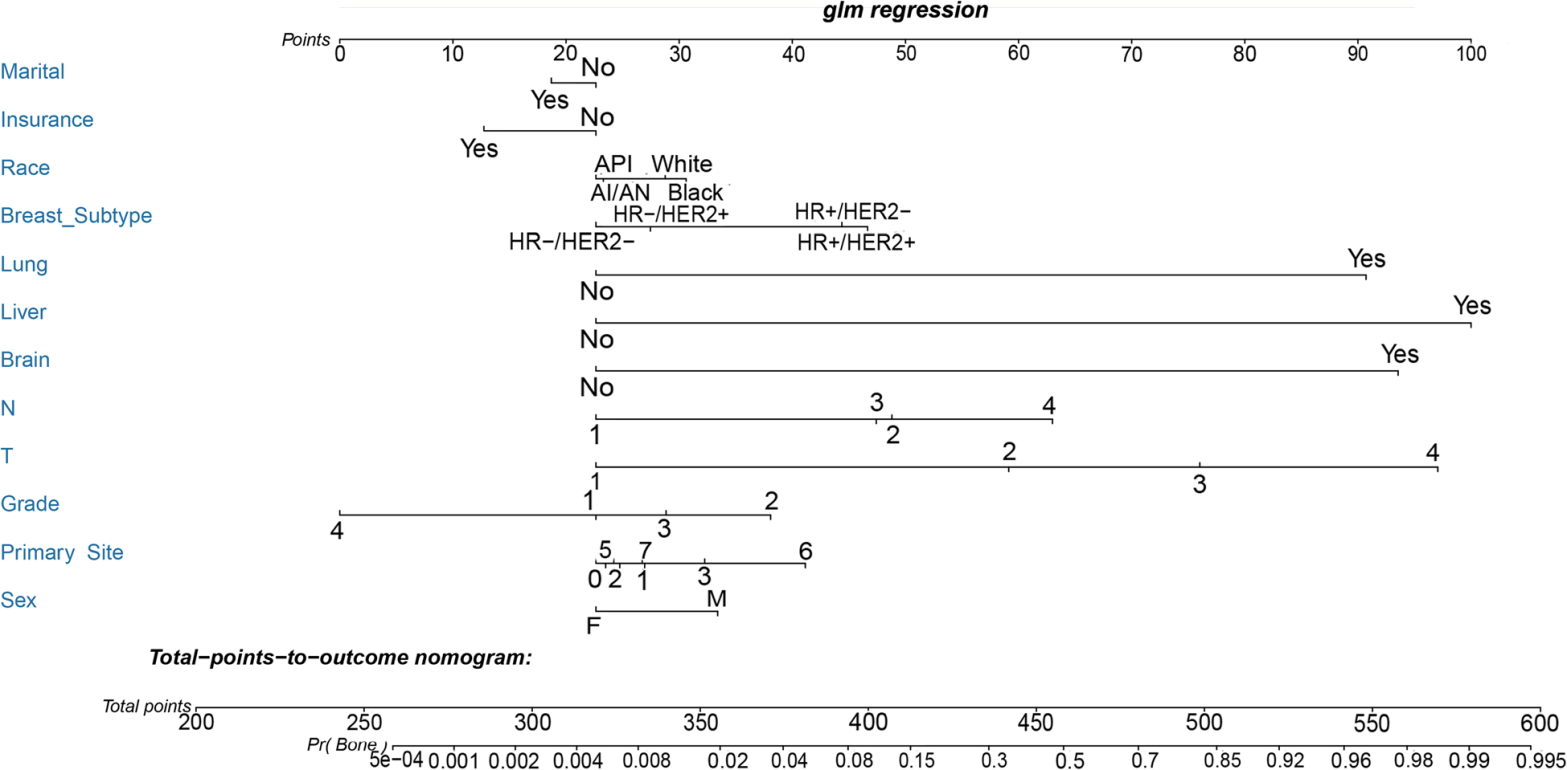
Nomogram to estimate the risk of BM in patients with IDC. In the diagnostic nomogram, values for the individual patient are located along the variable axes, and a line is drawn upward to the Points axis to determine the number of points assigned for each variable. There was a Total Points line at the bottom of the nomogram, and each variable score was summed to give the total points. Then, draw a vertical line from the total points scale to BM axis to obtain the probability

**Fig. 2:**
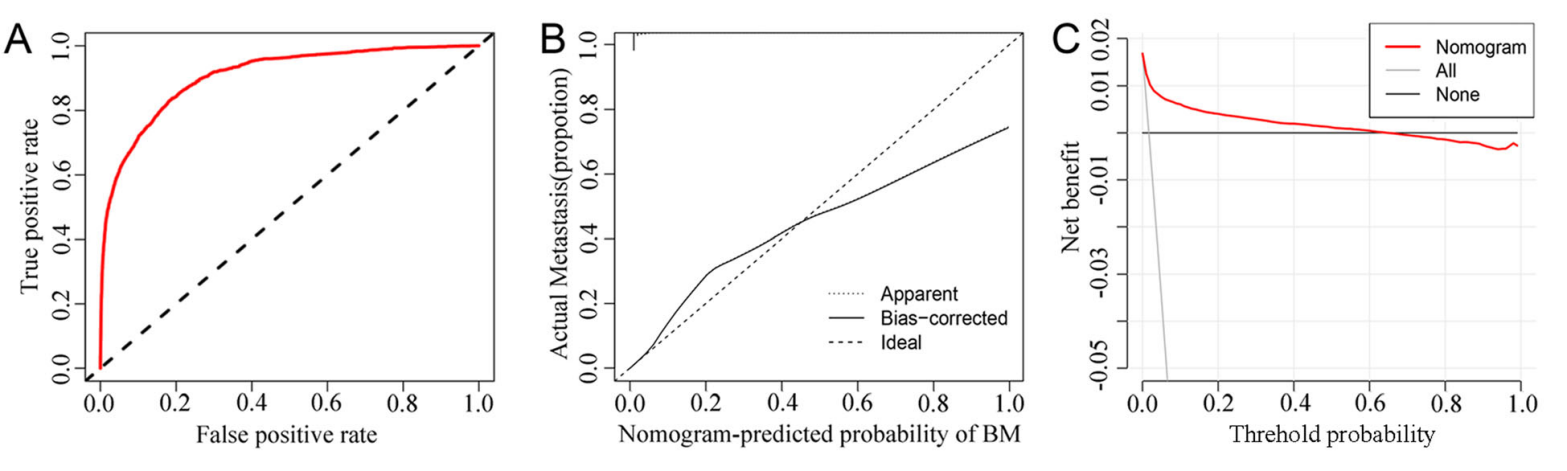
ROC curves, calibration curves and DCA of the diagnostic nomogram for estimating the risk of BM in patients with IDC. **a** The area under the ROC curve was used to show the discrimination of the diagnostic nomogram. **b** The X-axis represents the nomogram-predicted probability of BM; the Y-axis represents the actual probability of BM. Plots along the 45-degree line indicate a perfect calibration model in which the predicted probabilities are identical to the actual outcomes. **c** This diagnostic nomogram shows a notable positive net benefit, indicating that it has a good clinical utility in predicting estimating the risk of BM in patients with IDC

A nomogram for predicting the risk of BM in IDC patients was established based on the independent predictors (Fig. 1). ROC analysis showed that the AUCs of the nomogram reached 0.907, demonstrating a better discriminative ability (Fig. 2a). The calibration curve showed high consistency between the observed and predicted results (Fig. 2b). In addition, the DCA indicated that the nomogram had good performance in clinical practice (Fig. 2c).

### Prognostic factors for IDC patients with BM

In the training cohort, the univariate Cox proportional hazards regression analysis showed that age, race, primary site, grade, radiotherapy, surgery, chemotherapy, liver metastasis, lung metastasis, brain metastasis, breast cancer subtype, HER2 status, insurance status, and marital status were prognostic factors (all *P* < 0.05) (Table 5). Then, the multivariate Cox proportional hazards regression analysis was performed. Finally, ten factors, including age, race, grade, surgery, chemotherapy, brain metastases, liver metastases, breast cancer subtypes, insurance status, and marital status, were identified as independent prognostic factors for OS (Table 5).

**Table 5:** Univariate and multivariate Cox proportional hazards regression analysis in infiltrating duct carcinoma patients with bone metastasis

Characteristics	Univariate analysis		Multivariate analysis	
	HR (95% CI) *P* value		HR (95% CI) *P* value	
Race				
Black	Reference		Reference	
Other	0.504 (0.359–0.707)	< 0.001	0.547 (0.387–0.772)	0.001
White	0.695 (0.579–0.834)	< 0.001	0.686 (0.567–0.831)	< 0.001
Age				
22–54	Reference		Reference	
55–79	1.511 (1.296–1.763)	< 0.001	1.523 (1.302–1.782)	< 0.001
≥ 80	2.414 (1.942–3.001)	< 0.001	2.241 (1.768–2.841)	< 0.001
Sex				
Female	Reference			
Male	1.236 (0.801–1.906)	0.338		
Primary Site				
Nipple	Reference			
Central portion of breast	0.443 (0.204–0.961)	0.039		
Upper-inner quadrant of breast	0.557 (0.258–1.205)	0.138		
Lower-inner quadrant of breast	0.619 (0.283–1.353)	0.229		
Upper-outer quadrant of breast	0.591 (0.279–1.251)	0.170		
Lower-outer quadrant of breast	0.468 (0.214–1.024)	0.057		
Axillary tail of breast	0.339 (0.099–1.159)	0.085		
Overlapping lesion of breast	0.536 (0.253–1.139)	0.105		
Grade				
I	Reference		Reference	
II	1.658 (1.161–2.368)	0.005	1.902 (1.329–2.721)	< 0.001
III	2.436 (1.710–3.470)	< 0.001	2.819 (1.958–4.057)	< 0.001
IV	4.156 (1.274–13.557)	0.018	2.527 (0.761–8.395)	0.13
Laterality				
Left - origin of primary	Reference			
Right - origin of primary	1.022 (0.893–1.169)	0.752		
T stage				
T1	Reference			
T2	0.945 (0.765–1.167)	0.600		
T3	1.252 (0.988–1.585)	0.063		
T4	1.237 (0.993–1.541)	0.058		
N stage				
N0	Reference			
N1	0.944 (0.796–1.120)	0.511		
N2	0.916 (0.726–1.155)	0.459		
N3	1.087 (0.875–1.350)	0.451		
Radiotherapy				
No	Reference			
Yes	0.735 (0.628–0.861)	< 0.001		
Surgery				
No	Reference		Reference	
Yes	0.562 (0.487–0.648)	< 0.001	0.575 (0.493–0.669)	< 0.001
Chemotherapy				
No	Reference		Reference	
Yes	0.769 (0.672–0.880)	< 0.001	0.730 (0.619–0.860)	< 0.001
Brain metastasis				
No	Reference		Reference	
Yes	2.721 (2.132–3.473)	< 0.001	2.189 (1.699–2.820)	< 0.001
Liver metastasis				
No	Reference		Reference	
Yes	1.851 (1.584–2.163)	< 0.001	1.744 (1.471–2.067)	< 0.001
Lung metastasis				
No	Reference			
Yes	1.535 (1.325–1.778)	< 0.001		
Breast subtype				
HR−/HER2-	Reference		Reference	
HR−/HER2+	0.263 (0.186–0.372)	< 0.001	0.281 (0.198–0.399)	< 0.001
HR+/HER2-	0.337 (0.278–0.409)	< 0.001	0.376 (0.299–0.474)	< 0.001
HR+/HER2+	0.304 (0.238–0.388)	< 0.001	0.312 (0.242–0.402)	< 0.001
HER2 status				
Positive	Reference			
Negative	1.322 (1.118–1.563)	=0.001		
Insurance				
Uninsured	Reference		Reference	
Insured	0.684 (0.519–0.902)	0.007	0.726 (0.545–0.966)	0.028
Marital status				
Unmarried	Reference		Reference	
Married	0.757 (0.662–0.866)	< 0.001	0.860 (0.748–0.989)	0.035

### Prognostic nomogram development and validation

**Fig. 3:**
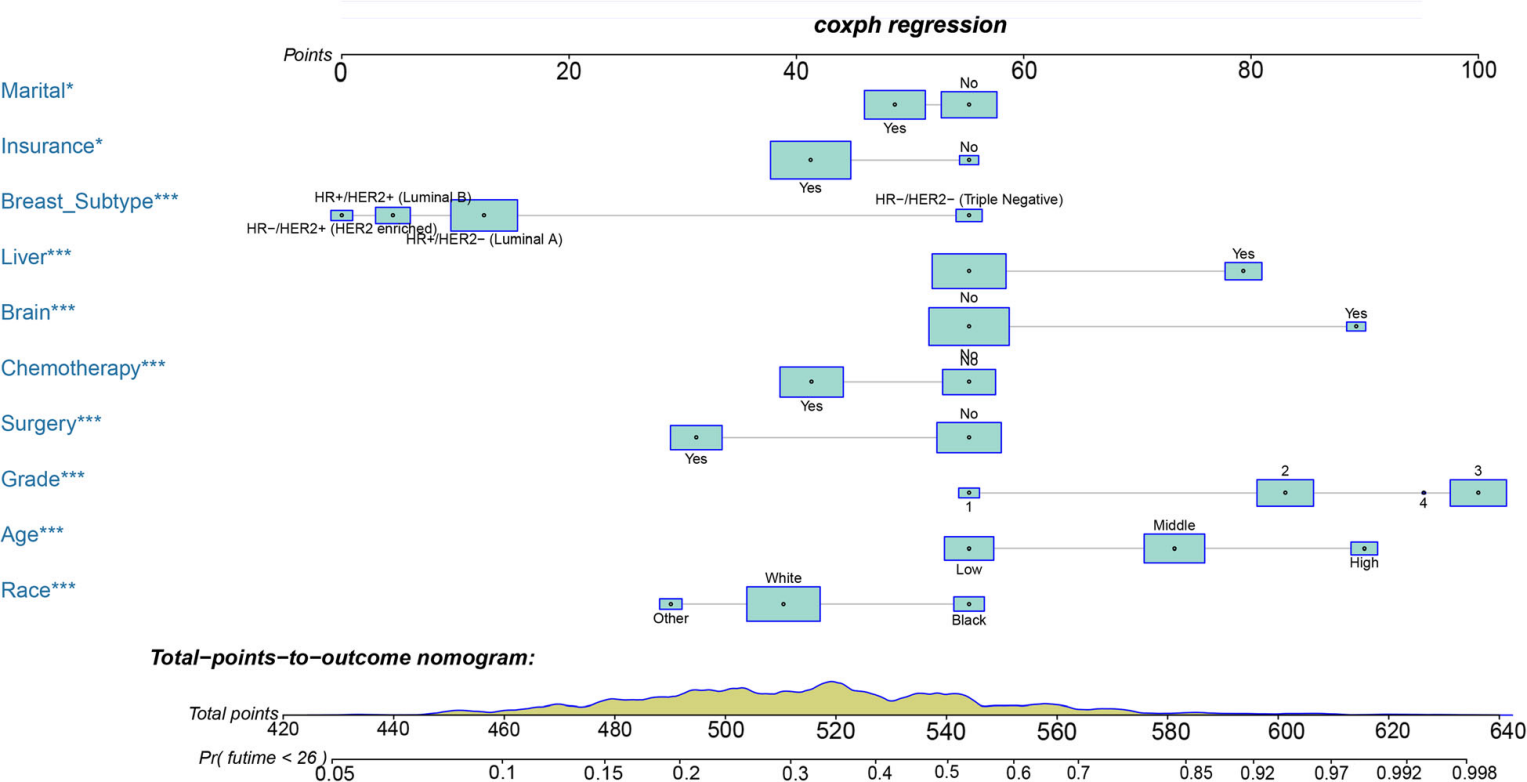
Nomogram to predict the survival of IDC patients with BM. In the prognostic nomogram, values for the individual patient are located along the variable axes, and a line is drawn upward to the Points axis to determine the number of points assigned for each variable. There was a Total Points line at the bottom of the nomogram, and each variable score was summed to give the total points. And the accumulated total points can be used to predict the 1-, 3-, and 5-year survival rate of the patient

**Fig. 4:**
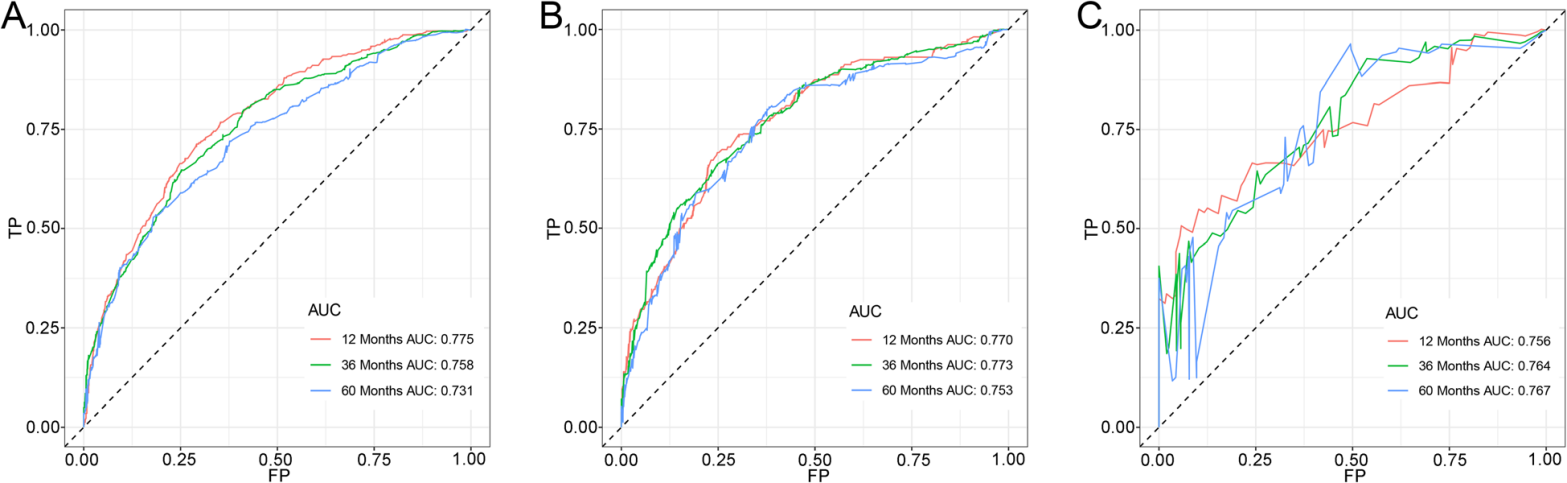
ROC curves of the nomogram in predicting prognosis at the 1-, 3-, and 5-year points in the training cohort (**a**), internal validation cohort (**b**) and external validation cohort (**c**). The corresponding time-dependent AUCs were used to show the discrimination of the prognostic nomogram. The red line represents the ROC curve for the prognostic nomogram in predicting the prognosis at the 1-year point. The green line represents the ROC curve for the prognostic nomogram in predicting the prognosis at the 3-year point. The blue line represents the ROC curve for the prognostic nomogram in predicting the prognosis at the 5-year point

**Fig. 5:**
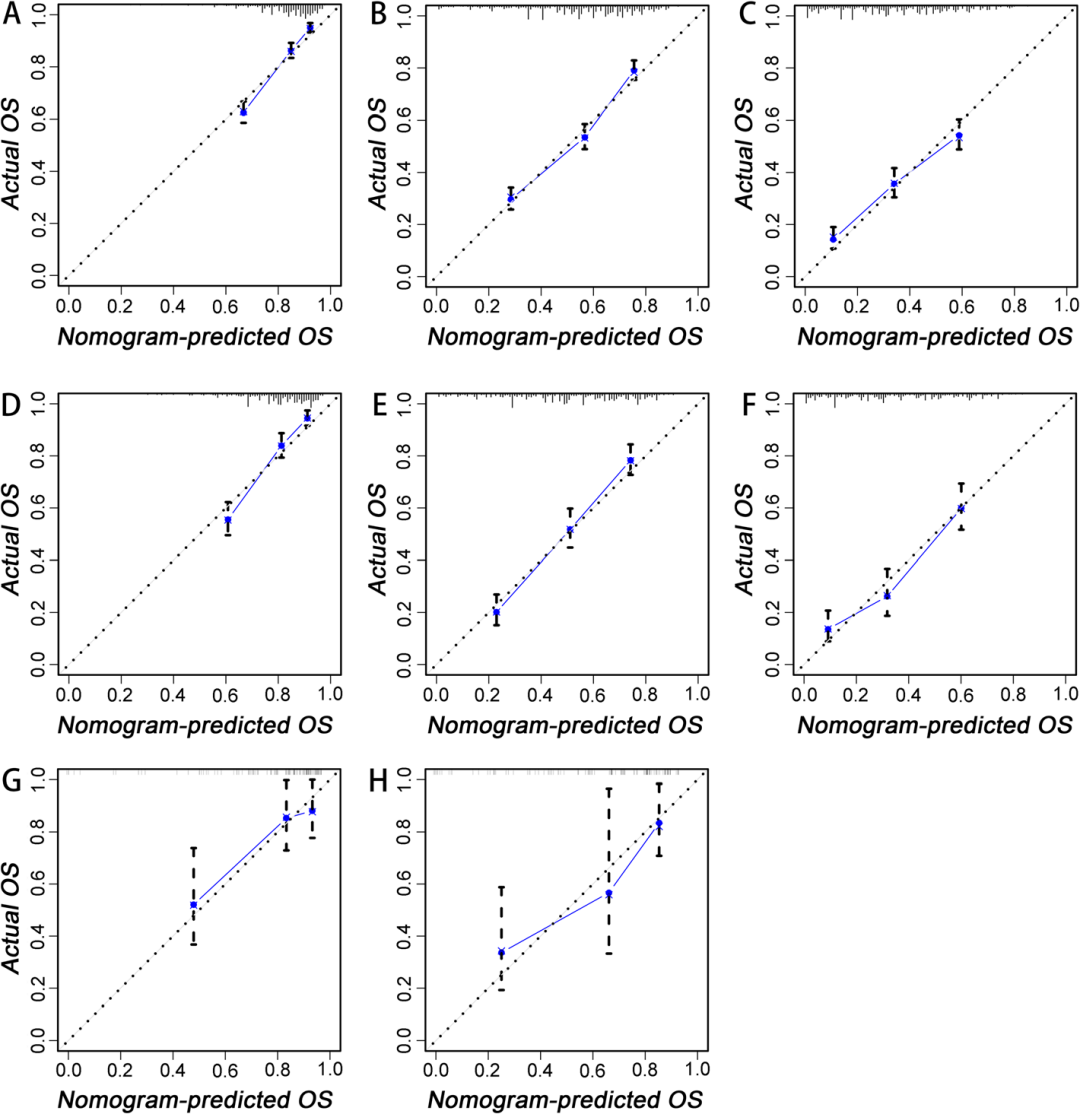
The calibration curves of the nomogram for the 1-, 3-, and 5-year OS prediction of the training cohort (**a**–**c**), internal validation cohort (**d**–**f**) and external validation cohort (**g**, **h**). The X-axis represents the nomogram-predicted OS probability; the Y-axis represents the actual OS probability. Plots along the 45-degree line indicate a perfect calibration model in which the predicted probabilities are identical to the actual outcomes. Vertical bars indicate 95% confidence intervals

**Fig. 6:**
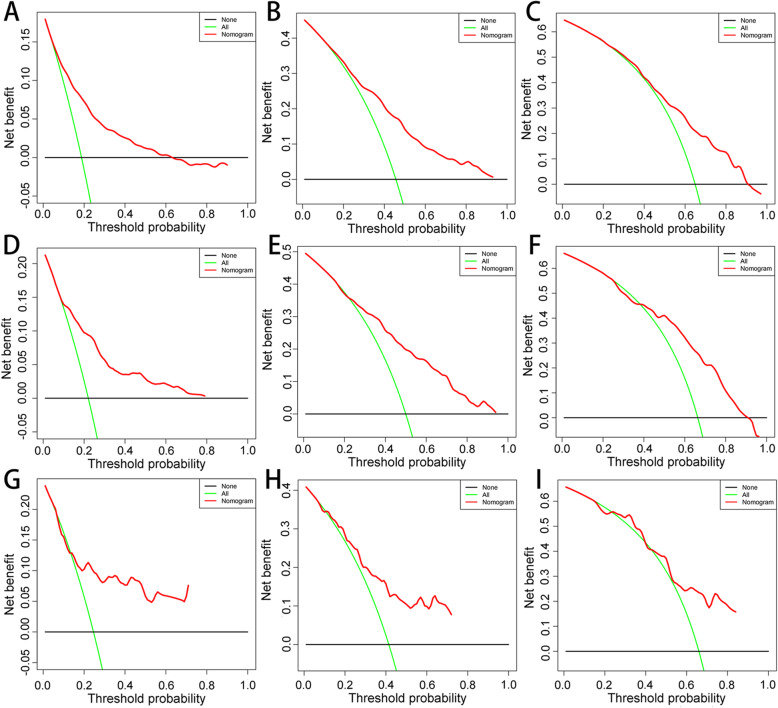
DCA of the nomogram for predicting the 1- (**a**), 3- (**b**) and 5- year (**c**) OS in the training cohort, the 1 (**d**), 3 (**e**) and 5-year (**f**) OS in the internal validation cohort and the 1 (**g**), 3 (**h**) and 5-year (**i**) OS in the external validation cohort. The x-axis is the threshold probability, the y-axis is the net benefit rate. The black horizontal line indicates that death occurred in no patients. The green oblique line indicates that all patients will have specific death. The red line represents the prognostic nomogram

**Fig. 7:**
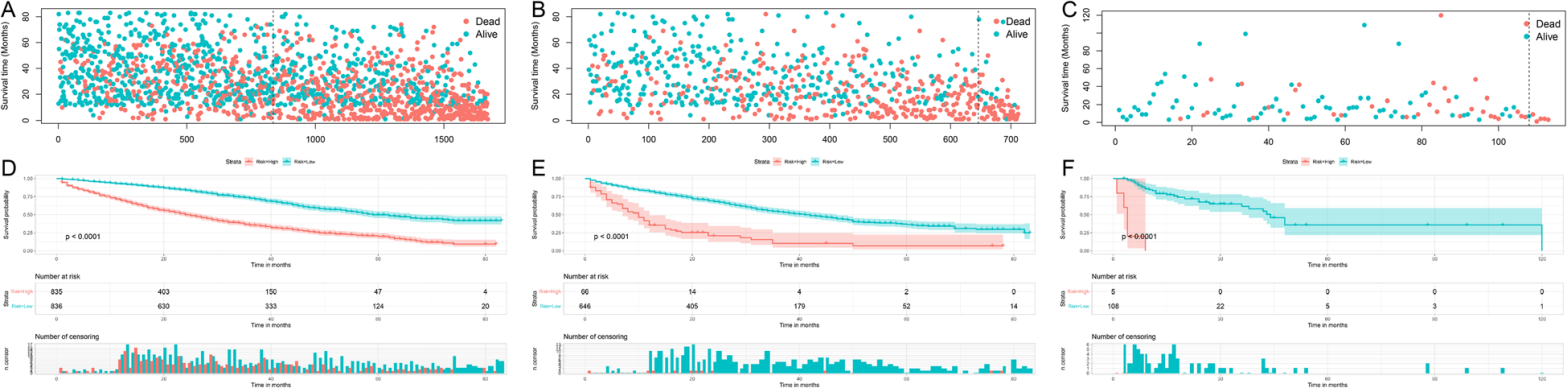
Kaplan–Meier survival analysis of the signature for both the training cohort and the validation cohort. In Kaplan–Meier survival analysis, red curve represents the subgroup with higher risk score, and green curve represents lower risk score. Patients with a high risk score demonstrated a worse OS than those with a low risk score in the training cohort (**a**, **d**), internal validation cohort (**b**, **e**) and external validation cohort (**c**, **f**), which suggests the strong predictive ability for BM patient survival outcome

Based on the prognostic factors selected in the training cohort, a nomogram was established to predict the OS for IDC patients with BM (Fig. 3). ROC analysis showed that the AUCs of these nomograms for the 1-, 3-, and 5-year OS reached 0.775, 0.758, and 0.731 in the training cohort; 0.770, 0.773, and 0.753 in the internal validation cohort; and 0.756, 0.764, and 0.767 in the external validation cohort, respectively (Fig. 4a, b, c). The calibration curves of the nomograms showed a strong agreement between actual observations and predictions (Fig. 5). Due to data reasons, the 5-year OS calibration curve for the AHOCMU cohort could not be generated. The clinical application value of the nomogram was evaluated by DCA. As shown in Fig. 6, this nomogram shows a notable positive net benefit over a wide range of death risks, indicating that it has a good clinical utility in predicting the OS for IDC patients with BM. The external validation using the established nomogram in the AHOCMU cohort also demonstrated the high accuracy of the prediction model. Kaplan–Meier survival analysis was performed on the training cohort, internal validation cohort, and external validation cohort, and the results showed that there was an obvious difference in survival rates between the three cohorts (Fig. 7).

## Discussion

Almost all deaths in patients with BC are caused by metastatic disease [4, 5]. Common metastatic sites include bone, lung, liver, and brain, of which bone is the most common [17, 18]. However, unlike the metastases to the lung, liver and brain, BM is generally considered to be less fatal [19]. Once BC patients are diagnosed with BM, the OS decreases dramatically and the median life expectancy decreases to 2–3 years [20, 21]. IDC is the most common pathological type of BC; therefore, it is necessary to identify the risk and prognostic factors for IDC patients with BM to facilitate the early prevention and detection of BM and improve the prognosis for IDC patients with BM.

Currently, there are many studies focused on BM in patients with BC, but there are few studies on IDC patients with BM. Chen et al. reported that in axillary lymph node metastasis, CA125, CA153, ALP, and hemoglobin concentration were independent risk factors for BM in BC patients [22]. Yue Gong et al. determined that age, ethnicity, histology, grade, tumor subtype, extra bone metastasis site, and education level were predictors of BM in BC patients [23]. Other studies have also reported that involvement of more than four axillary lymph nodes at initial diagnosis, primary tumor size greater than 2 cm, estrogen receptor positive and progesterone receptor negative tumors and younger age are risk factors for BM in BC patients [24, 25]. This is similar to the results of our study. In our study, sex, primary site, grade, T stage, N stage, brain metastasis, lung metastasis, liver metastasis, breast cancer subtype, race, insurance status and marital status were significant predictors for BM in IDC patients. Although Zhao et al. established a nomogram model based on gene expression to predict the risk of BM in BC patients, it is not suitable for a wide range of clinical applications and includes all types of BC, which is not conducive to individualized and accurate predictions [26]. To date, no realistic model has been established to predict the risk and prognosis of BM in ICD patients. To address this problem we extracted, screened, and organized specific and relevant prognostic and risk factors of IDC patients with BM and established an intuitive and practical prediction model. This model is beneficial to both the clinician and the individual patient.

It is generally believed that IDC with only metastases to the bone has a better OS prognosis than IDC with bone and visceral metastasis [27]. Previous studies have also found that patients with BM alone had a median survival of approximately two to three times that of patients with additional visceral metastases [28–30]. Lobbezoo DJ et al. compared the results of 815 patients with primary or recurrent metastatic BC and found that patients with visceral metastases and patients with multiple metastatic sites had a worse prognosis [31]. Interestingly, our results showed that the presence of brain metastasis and liver metastasis had a significant negative impact on the OS, which is consistent with the above results. In addition, we found that the number of metastatic organ sites also had a significant effect on survival. Previous studies have shown that patients with four metastatic sites are 2.2 times more likely to die than patients with only one metastatic site [27]. We speculate that patients with only BM develop vital organ dysfunction later, so these patients have a higher survival rate than those with both bone and extraosseous metastases. According to previous research, the breast cancer subtype is an independent risk factor for the occurrence of metastasis, and the incidence of BM is highest in BC patients that are HR+/HER2− or HR+/HER2+ [23, 32]. Our results show that patients with HR+/HER2- BC have a higher risk of BM, and patients with Grade 2 BC are more likely to have BM compared to patients with Grades 3 and 4 BC, which is controversial. At present, most people think that once a tumor has distant organ metastasis, it may accelerate the metastasis to other organs, which is consistent with our results [33]. According to our results, chemotherapy had a positive effect on prognosis. Contrary to what we expected, radiotherapy was not a relevant factor for prognosis. Unfortunately, we were unable to compare the effects of different chemotherapy regimens on survival rates because there was no detailed information on chemotherapy strategies in our data.

To facilitate clinical work, we established two nomograms to predict the risk and prognosis for BM in IDC patients. Through calibration, ROC curve and DCA, the nomogram shows great performance, both internally and externally, for predicting the prognosis of IDC patients with BM. These models have better prediction capabilities and higher credibility and can provide references for patient consultations, risk assessment and clinical decision-making. To our knowledge, this is the first population-based model to predict the risk and prognosis of newly diagnosed BM in IDC patients. However, we should acknowledge that this study has some limitations. First, it is a retrospective study and only patients with complete information were included. Therefore, selection bias is likely to exist. Second, some patients with BM have no symptoms, causing the number of newly diagnosed patients with BM to be lower than the actual number. Third, we did not have specific information about systemic treatments, such as endocrine therapy or HER2 targeted therapy. Fourth, since the data in this study were from the SEER database, the nomogram we constructed may not be applicable to IDC patients worldwide.

## Conclusion

These nomograms could be used as a supportive graphic tool in IDC to help clinicians distinguish, assess and evaluate the risk and prognosis of IDC with BM. Internal and external validation and application in an independent population demonstrated the satisfactory performance and clinical utility of this predictive model.

## Data Availability

The dataset from SEER database generated and/or analyzed during the current study are available in the SEER dataset repository (https://seer.cancer.gov/).

## Abbreviations

BC: Breast cancer
BM: Bone metastasis
AJCC: American Joint Committee for Cancer
IDC: Infiltrating duct carcinoma
SEER: Surveillance, epidemiology and end results
ER: Estrogen receptor
PR: Progesterone receptor
AHOCMU: Affiliated Hospital of Chengde Medical University
OS: Overall survival
ROC: Receiver operating characteristic
AUC: Area under the curve
DCA: Decision curve analyses
